# MicroRNA Regulation of Breast Cancer Stemness

**DOI:** 10.3390/ijms22073756

**Published:** 2021-04-04

**Authors:** Brock Humphries, Zhishan Wang, Chengfeng Yang

**Affiliations:** 1 Center for Molecular Imaging, Department of Radiology, University of Michigan, 109 Zina Pitcher Place, Ann Arbor, MI 48109, USA; 2 Division of Cancer Biology, Department of Medicine, MetroHealth Medical Center, Case Western Reserve University School of Medicine, 2500 MetroHealth Drive, Cleveland, OH 44109, USA; zhishan.wang@case.edu

**Keywords:** cancer stem-like cell, tumor-initiating cell, microRNA, breast cancer, stemness

## Abstract

Recent advances in our understanding of breast cancer have demonstrated that cancer stem-like cells (CSCs, also known as tumor-initiating cell (TICs)) are central for progression and recurrence. CSCs are a small subpopulation of cells present in breast tumors that contribute to growth, metastasis, therapy resistance, and recurrence, leading to poor clinical outcome. Data have shown that cancer cells can gain characteristics of CSCs, or stemness, through alterations in key signaling pathways. The dysregulation of miRNA expression and signaling have been well-documented in cancer, and recent studies have shown that miRNAs are associated with breast cancer initiation, progression, and recurrence through regulating CSC characteristics. More specifically, miRNAs directly target central signaling nodes within pathways that can drive the formation, maintenance, and even inhibition of the CSC population. This review aims to summarize these research findings specifically in the context of breast cancer. This review also discusses miRNAs as biomarkers and promising clinical therapeutics, and presents a comprehensive summary of currently validated targets involved in CSC-specific signaling pathways in breast cancer.

## 1. Introduction

Breast cancer is the most common occurring form of cancer in women and the second most common cancer in the world [1]. Breast cancer is also the second leading cause of cancer-related death in women, highlighting the urgent need to better understand the underlying mechanisms that drive breast cancer initiation and progression. One of the major underlying causes of breast cancer mortality is recurrence, or cancer that comes back after a period of time when the cancer could no longer be clinically detected. When recurrence occurs at the same location as the primary tumor, it is called local recurrence. However, if the cancer has spread to organs or tissues of the body far from the original site of cancer, then it is termed distant or metastatic recurrence. Interestingly, distinct differences exist between breast cancer subtype recurrence rates and correlate well with the hormone receptor levels in breast cancer [2,3,4], demonstrating that subtype is important and should be taken into account for patient recurrence rates.

Accumulating evidence demonstrates that the acquisition of stem-like features is a major contributor to breast cancer recurrence and metastasis. This suggests that identifying mechanisms that control cancer stemness can elucidate novel therapies for the treatment of breast cancer. In this review, we focused on recent exciting findings showing that microRNAs are critical in regulating stemness to ultimately control breast cancer recurrence and progression. We aimed to summarize the specific signaling pathways that contribute to the acquisition of cancer stem-like characteristics as well as the outlook for the use of microRNAs in the clinic as prognostic and diagnostic markers and as a therapeutic intervention.

## 2. Cancer Stem Cells and Breast Cancer Recurrence

Cellular heterogeneity within tumors is a major underlying cause for recurrence. There are two models that are currently used to explain breast cancer heterogeneity [5,6]. The first is the clonal evolution model, which focuses on cells acquiring random mutations and subsequent clonal selection. The other is the cancer stem cell (CSC) model, which posits that tumors are hierarchically organized and at the apex of this hierarchy are cells that display stem cell properties. Under normal physiological conditions, stem cells (SCs) are critical for maintaining tissue homeostasis and repair after injuries. SCs are a limited subpopulation of each tissue and are defined by having the capacity for unlimited self-renewal, as well as the ability to proliferate and produce all of the more differentiated cells that form tissues. Additionally, SCs cannot survive outside of their environment or in the absence of certain growth factors or cytokines [7,8], and they require these growth factors or cytokines to maintain the SC population [9,10]. However, as cells undergo malignant transformation, they can also obtain these properties/functions and, contrary to the physiological role of SCs, utilize them to support cancer initiation, maintenance, progression, and recurrence. These stem-like characteristics not only include unlimited self-renewal and the ability to give rise to a heterogeneous population of cells similar to the primary tumor with high plasticity [11,12,13,14,15], but they are also more resistant to an environmental reduction of oxygen and/or nutrients as well as to cancer therapy [16,17]. As one of the functionally defining characteristics of a CSC is the ability to form a tumor, CSCs have also been known as tumor-initiating cells (TICs).

## 3. Molecular Markers and Functional Methods to Identify and Characterize Breast CSCs

Currently, the acquisition of stem-like characteristics is defined either through the identification of surface or internal markers, or they are defined through functional analyses (Table 1). Among these molecular markers, the use of aldehyde dehydrogenase 1 (ALDH1^+^, measured by an ALDEFLUOR assay [18]) and CD24^−/low^/CD44^+^ [19] are the most commonly used to identify breast CSCs in research. Early reports on these molecular markers demonstrated that small numbers of cells exhibiting either of these molecular markers have increased tumor-initiating capacity in mice [19]. Interestingly, molecular profiling of these CSC populations demonstrate that they are distinct from one another [20,21]. The CD24^−/low^/CD44^+^ population marks a more mesenchymal, quiescent population of breast CSCs (also known as epithelial–mesenchymal transition (EMT)-CSCs), while ALDH1^+^ cells demarks an epithelial, proliferative subtype of breast CSCs (also known as mesenchymal–epithelial transition (MET)-CSCs) [20]. As breast cancer cells can transition between EMT and MET states and these transitions generate breast CSCs [22,23], it is postulated that breast CSCs also can transition between these two subtypes. Indeed, it was recently shown that these two subpopulations of breast CSCs are plastic and transition between these states [20,24,25], adding an underlying complexity to this dynamic process. Further work has identified molecular mechanisms that control these breast CSC states [25], elucidating potential therapeutic targets for these cells.

Outside of ALDH1 and CD24/CD44, many other breast CSC markers have been identified using cell lines or mouse models (Table 1). In cell lines, breast CSCs tend to exhibit, in alphabetical order, ABCG2^+^, CD24^−/low^/CD44^+^/ANTXR1^+^, CD24^−/low^/CD44^+^/SSEA-3^+^, CD49f^+^/DLL1^hi^/DNER^hi^, CD70^+^, ESA^hi^/PROCR^hi^/SSEA-3^+^, GD2^+^, Lgr5^hi^, MUC1^+^, Nectin-4^+^, or Procr^+^/ESA^+^ phenotypes [43]. Meanwhile, in mouse models or patient-derived xenograft (PDX) tumors, breast CSCs exhibit CD24^+^/Thy1^+^, CD24^+^/CD29^+^/CD49f^+^, CD29^low^/CD24^+^/CD61^+^, CD133^+^, or Sca1^+^ phenotypes [43]. The differences in CSC markers identified among model systems may be explained by the dynamic states of these cells, or CSCs may exhibit multiple markers that are unique to them compared to the bulk tumor. However, more work is needed to clarify which markers definitively identify breast CSCs.

In addition to molecular markers, the acquisition of SC phenotypes can be functionally defined. These functional assays capitalize on the major properties that define breast CSCs (Table 2) and allow for the analysis of effects on breast CSCs and in some cases retrieval for further downstream analysis. Examples of these assays include clonogenicity (soft agar colony formation) [44], tumor-initiating capacity (sphere formation (in vitro) or limiting dilution (in vivo)) [45,46], migration [21], dye-retention assays [34,47], and chemoresistance (drug curves or identification of the side population) [48,49]. While the use of one of these assays helps define effects on breast CSCs, the combination of many of these assays is critical for the definitive identification of CSCs.

### MicroRNAs and Breast CSCs

It came as a surprise that only about 1.5% of the human genome encodes the known ≈21,000 distinct protein-coding genes [50]. However, the other 98.5% of the human genome is not “junk” but is instead transcribed into RNA for non-protein-coding genes (ncRNA), if even at a low level [51,52]. These ncRNAs perform many specific functions within the cell including regulating RNA splicing, translation, DNA replication, and signal transduction, among others. Of the ncRNAs, microRNAs (miRNAs) are of particular interest due to their essential involvement in cancer initiation and progression. MiRNAs are a large (over 2600 [53]) family of endogenous, small (mature miRNA range from 18–25 nucleotides) RNAs that exclusively negatively regulate protein-coding gene expression in both metazoans and plants [54]. Biologically, miRNAs have promoters, start sites, and can also be regulated by post-transcriptional modifications just as protein-coding genes [55,56,57]. MiRNA expression is primarily regulated through interactions with and modifications of their promoters. For example, hypermethylation of, or transcription factor binding to, the miRNA promoter region can directly regulate the transcription of the miRNA [58,59,60,61]. Additionally, after transcription, miRNA function can be repressed by interactions with lncRNAs that sequester miRNAs [62]. They can also be packaged into extracellular vesicles and released to be picked up by other cells or to circulate freely in the body, permitting the use of miRNAs as potent biomarkers for the diagnosis and prognosis of breast cancer.

As miRNAs are predicted to target over 60% of the human protein-coding genes at the post-transcriptional level [63,64,65,66,67], miRNAs are implicated in almost all cellular functions. Since the acquisition of stem-like characteristics has emerged as an important factor in driving cancer initiation, progression, and recurrence, microRNAs likely are involved in regulating pathways that inhibit or allow cancer cells to obtain stem-like phenotypes. Indeed, a recent study demonstrated that the suppression of DICER, an enzyme critical for the microprocessing of miRNAs [68,69,70], by hypoxia is critical for the acquisition of stem cell properties in breast cancer [71], and a growing body of evidence shows that miRNAs occupy a prominent place in generating, maintaining, and in the propagation of breast CSCs [72]. More specifically, miRNAs play a more decisive role in breast CSCs by directly targeting central nodes within CSC-dependent signaling pathways, including Wingless and Int-1 (Wnt)/β-catenin, Janus kinase/signal transducer and activator of transcription (JAK/STAT), and Notch, among others. Thus, the goal of this review is to summarize recent exciting research showing how microRNAs regulate breast cancer stemness through these important signaling pathways and discuss its implications in developing new breast cancer therapies.

## 4. MiRNA Regulation of Key Signaling Pathways That Mediate Breast CSCs

The molecular signaling pathways that govern normal SC homeostasis are tightly regulated. Therefore, it is not surprising that many of these pathways are abnormally activated or repressed in breast CSCs [43,73]. Among these many pathways, Wnt/β-catenin, JAK/STAT, Notch, B lymphoma Mo-MLV insertion region 1 homolog (BMI1), Transforming growth factor beta (TGF-β), phosphoinositide 3-kinase (PI3K)/Akt and nuclear factor kappa-light-chain-enhancer of activated B cells (NF-κB) represent major signaling pathways that contribute to CSC physiology. In addition to acting independently, these pathways also converge on common downstream transcription factors, and many of these pathways are interwoven, which creates feedback loops. This section will focus on miRNAs that have been identified to promote or repress breast CSC phenotypes by specifically acting on the key signaling nodes involved in these pathways. A full list of the miRNAs that have been shown to regulate phenotypes of CSCs and the targets of those miRNAs for these specific pathways is found in Table 3.

### 4.1. Wnt/β-Catenin Signaling Pathway

The Wingless and Int-1 (Wnt)/β-catenin signaling pathway is critical for embryonic development and tissue homeostasis, and aberrant signaling facilitates cancer stem cell renewal, proliferation, and differentiation [141,142]. In this pathway, the cysteine-rich Wnt glycoproteins are secreted by cells into the extracellular matrix and activate receptor-mediated signaling upon binding to the Frizzled family of G-protein-coupled receptors. This activation disrupts the destruction of β-catenin (though a complex formed by axin, adenomatous polypoisis coli (APC), casein kinase 1α (CK1α), and glycogen synthase kinase 3β (GSK3β) [143]), driving the accumulation of β-catenin in the nucleus, where it interacts with the T cell factor/lymphoid enhancer factor-1 (TCF/Lef1) transcription complex to drive Wnt-mediated gene transcription. Typically, abnormal regulation of β-catenin leads to the acquisition of CSC phenotypes. As miRNAs are known to be important for the tight regulation of signaling pathways, it is no surprise that miRNAs have been shown to regulate the mediators of Wnt/β-catenin signaling to modulate the acquisition of CSC phenotypes.

As the driving force of this signaling pathway, miRNA targeting of Wnt ligands can have significant biological effects on CSCs. Lo et al. found that NEAT1, a long ncRNA, was regulated by BRCA1 and was important for breast tumorigenesis [30]. To further determine the mechanism for how NEAT1 drives breast tumorigenesis, this group utilized a miRNA screen and found that miR-129-5p was significantly upregulated in NEAT1-knockdown cells. The expression of miR-129-5p reduced both CD24^−^/CD49f^+^/CD44^+^ and CD24^−^/CD49f^+^/CD44^+^/EpCAM^+^ breast CSC populations, and, as a functional test, they found that the expression of miR-129-5p reduced the ability of MCF10A and MCF10DCIS cells to form spheres and grow in soft agar. This group further elucidated that miR-129-5p regulates breast CSCs through direct targeting of Wnt4. Exogenous treatment of miR-129-5p stably expressing cells with Wnt4 was able to overcome the inhibitory role of miR-129-5p on breast stemness [30]. This demonstrates the critical role of miR-129-5p on breast CSC homeostasis. In support of this, other miRNAs have also been shown to target other Wnt ligands in breast cancer [90,115].

In addition to regulating the Wnt ligands, miRNAs can also directly target the Frizzled receptors. Liu and colleagues isolated CSCs (using ESA^+^/CD24^−^/CD44^+^/lin^−^ CSC and ESA^+^/CD24^+^/CD44^−^/lin^−^ non-CSC markers) from six breast cancer tissue samples and identified any changes in the miRNAs by miRNA microarray [74]. From this, miR-1 expression was found to be downregulated in breast CSCs, and further analysis discovered that miR-1 negatively associated with the more aggressive subtypes of breast cancer. To better understand the function of miR-1 in breast CSCs, the expression level of miR-1 was manipulated in both MCF-7 and SK-BR-3 breast cancer cell lines. Analysis of CD24/CD44 breast CSCs markers showed that inhibition of miR-1 increased, while overexpression of miR-1 decreased CD24^−^/CD44^+^ CSC markers in both MCF7 and SK-BR-3 cells. Furthermore, separating cells into CD24^−^/CD44^+^ CSC and CD24^+^/CD44^−^ non-CSC populations demonstrated that miR-1 levels were significantly higher in the non-CSC population than the CSC population. Looking specifically at Wnt/β-catenin signaling, they found that the transfection of breast cancer cells with miR-1 significantly inhibited both protein levels and luciferase reporter activity of the Frizzled 7 receptor, demonstrating that the Frizzled 7 receptor is a direct target of miR-1 [74]. Furthermore, these cells also had significantly reduced ratios of nuclear to cytosolic β-catenin. Together, these data show that miR-1 reduces the breast CSC population by targeting the Wnt/β-catenin pathway.

Interestingly, miRNAs can also target key proteins involved in the destruction of β-catenin. Feliciano and colleagues found that miR-125b directly targets casein kinase 2α (CK2-α) to regulate functions of breast CSCs [103]. Using a miRNA microarray, they found 939 differentially regulated miRNAs between breast cancer tumor samples and normal breast tissue, with miR-125b the only miRNA to be significantly downregulated in breast cancer and able to distinguish tumors from normal breast tissue. Stable expression of miR-125b in MCF7 and MDA-MB-231 breast cancer cells resulted in a decreased ability of these cells to grow in soft agar, and this group found that miR-125b is able to accomplish this through direct targeting of CK2-α.

The miR-200 family has been implicated in many facets of breast cancer initiation and progression [57]. Therefore, it is not surprising that this family has also been shown to regulate breast CSCs. In support of this, Liu et al. found that two of the miR-200 family members, miR-200c and -141, regulate breast CSC homeostasis [120]. In a miR-200c/141 cluster deletion mouse model of breast cancer, they found that the knockout of miR-200c/141 resulted in an increase in the CD24^+^/CD29^+^ and a decrease in the ALDH^+^ CSC marker, suggesting that these miRNAs regulate breast CSCs. These data were repeated in SUM149 and T47D human breast cancer cells. Mechanistically, this group found that miR-200c/141 directly targeted HIPK1, a β-catenin interacting protein, and either inhibiting miR-200c/141 or overexpressing HIPK1 increased β-catenin activation [120]. Inhibition of β-catenin by ICG-001 in HIPK1 stably expressing cells partially inhibited the changes in breast CSC markers, suggesting that miR-200c/141 directly regulate breast CSC homeostasis not through Wnt ligands or their receptors but through the Wnt/β-catenin interactor HIPK1.

### 4.2. Notch Signaling Pathway

The Notch signaling pathway is a normal mammalian developmental pathway that is necessary for stem cell fate decisions and differentiation [144,145]. Ligands for the four Notch receptors (Notch1-4) include delta-like proteins 1, 3, and 4 (DLL1, 3, and 4) and the Jagged proteins (JAG1 and JAG2). Both ligands and receptors are transmembrane proteins; thus, the activation of Notch signaling occurs by cell-to-cell contact. Once bound, it initiates proteolytic cleavage (first by ADAM protease and then γ-secretase) of the intracellular domain of the Notch receptors, leading to a release and translocation of the intracellular domain into the nucleus. Once in the nucleus, the intracellular domain displaces co-repressors of, and binds to, the CBF1/Suppressor of Hairless/LAG1 (CSL)/RBPJ transcription factor to drive Notch-mediated signaling [145]. MicroRNAs have been shown to not only directly regulate the Notch receptors themselves but also other signaling components.

Studies have shown that miRNAs directly target Notch receptors to regulate breast CSCs. For example, multiple studies have demonstrated that the Notch1 receptor is critical for functions of breast CSCs [76,91,109]. These studies demonstrated that miR-34a, miR-9, and miR-139-5p are all downregulated in cancer compared to normal breast tissue or cells, and all are negatively correlated with Notch1 expression. In each of these studies, the miRNA was able to reduce breast cancer stemness markers [76,91] or sensitize breast cancer cells to chemotherapy [91,109], and this was accomplished in part due to the direct targeting of Notch1. Additionally, Chao and colleagues identified Notch2 as a direct target of miR-205, and they found that the stable expression of miR-205 or knockdown of Notch2 reduced CD24^−^/CD44^+^ CSC markers and diminished sphere formation [124]. Lastly, Yu et al. found that Notch4 was negatively regulated by miR-34c in breast TICs [93]. The expression of miR-34c or knockdown of Notch4 in breast TICs resulted in a decrease in their ability to form spheroids and a reduction of CD24^−/low^/CD44^+^ and ALDH^+^ breast CSC markers.

In addition to targeting the Notch receptors described above, miRNAs also target the Notch signaling pathway ligands. For example, Lee et al. analyzed miRNA changes in MCF-7 breast cancer cells in response to estrogen [92]. Of the estrogen-regulated miRNAs, miR-34b was one of the most highly downregulated miRNAs after treatment with estrogen, and miR-34b expression negatively correlates with estrogen receptor status in breast cancer tissues. Estrogen increased the ability of MCF-7 cells to grow in a colony formation assay, and miR-34b decreased this estrogen-dependent increase. It was later determined that miR-34b inhibits this growth by direct targeting of JAG1, and stable expression of miR-34b significantly reduced tumor growth in a mouse model of breast cancer [92]. In addition, Shui and colleagues also demonstrated that miR-130b-3p regulates functions of breast CSCs and breast cancer progression by targeting DLL1 [108]. These data show that miRNAs can regulate functions of breast CSCs by regulating Notch ligands.

### 4.3. JAK/STAT Signaling Pathway

In the Janus kinase/signal transducer and activator of transcription (JAK/STAT) pathway, tyrosine kinase JAKs are bound to the cytoplasmic regions of type I and II cytokine receptors [146,147,148]. Once cytokines bind to these receptors, the receptors homo- or heterodimerize, recruit JAKs, and these JAKs are then transphosphorylated, ultimately leading to recruitment of one or more STATs for phosphorylation. Phosphorylated STATs translocate to the nucleus, dependent on importin α-5, and either activate or repress the transcription of target genes [149,150]. There are a variety of ligands and receptors that stimulate the JAK/STAT signaling pathway, and there are a wide range of cytoplasmic proteins that function as regulators of this pathway, including miRNAs. A failure to regulate JAK/STAT signaling properly has been shown to drive a multitude of diseases, including cancer; thus, the regulation of JAK/STAT mediators by miRNAs is an important mechanism for the acquisition of stemness in breast cancer.

The majority of the research regarding miRNAs and the JAK/STAT pathway has shown that miRNAs regulate the various STAT proteins. Therefore, this section will focus on miRNAs and their STAT targets. Controversial results exist for the role of miRNAs in the regulation of STAT3 on CSC formation and maintenance. On one hand, multiple studies have shown that a decrease of STAT3 leads to the inhibition of CSCs. For example, comparing the miRNA expression levels of ALDH^+^ (CSCs) to ALDH^−^ (non-CSCs) breast cancer cells, Liu et al. found that miR-93 was significantly increased in the non-CSC population [94]. Furthermore, they found that the stable expression of miR-93 significantly decreased ALDH^+^ and CD24^−/low^/CD44^+^ CSC markers and significantly reduced their tumor-forming capabilities in mice due to the direct targeting of STAT3. In support of this study, other work has shown that miRNAs that directly target STAT3 result in a decrease not only in markers of breast CSCs but also the physiology of breast CSCs [134,135,137]. In contrast to these studies, Liu and colleagues found that extracellular matrix metalloproteinase inducer (EMMPRIN) drives the formation and maintenance of breast CSCs via an increase of CD44 and a decrease in CD24 markers through the induction of STAT3 [98]. They later found that EMMPRIN drives STAT3 through the downregulation of miR-106a/b, which directly targets STAT3.

In addition to STAT3, other STATs are also the targets of miRNAs. Using a bioinformatics approach, Pinatel and colleagues found that miR-223 expression was positively correlated with breast cancer progression [129]. However, when validated in breast cancer cells, miR-223 had extremely low endogenous expression in MDA-MB-231, MCF7, or T47D cells. Instead, they found that miR-223 was transferred from the stromal cells to the cancer cells. When miR-223 was stably expressed, these cells became more sensitive to commonly used chemotherapeutics, doxorubicin and paclitaxel, suggesting a tumor-suppressive role for miR-223 on breast CSCs. It was later discovered that miR-223 accomplishes this through the direct targeting of STAT5A [129]. The miR-200 family member, miR-141, has also been shown to directly target STAT5A in breast cancer [111]. Treating T47D breast cancer cells with progesterone resulted in a decrease in miR-141 expression levels, which is concurrent with an increase in CD44 expression and ability to form spheres. Further mechanistic studies revealed that STAT5A is a direct target of miR-141, and that STAT5A is critical for the acquisition of the CK5 breast CSC marker [111]. Lastly, Yan and colleagues found that STAT6 is a direct target of miR-1207-5p in breast cancer, and the downregulation of STAT6 by miR-1207-5p is critical for the inhibition of clonogenicity of these cells [101].

### 4.4. PI3K and PTEN Signaling Pathway

In response to ligands binding to their corresponding receptor tyrosine kinases (RTKs), cytoplasmic PI3K phosphorylates phosphatidylinositol (3,4)-bis-phosphate (PIP_2_) to become phosphatidylinositol (3,4,5)-bis-phosphate (PIP_3_), providing a docking site for Akt, also known as protein kinase B (PKB). Once bound to PIP_3_ at the plasma membrane, Akt is phosphorylated at T308 by phosphoinositide-dependent kinase 1 (PDK1) for partial activation and at S473 by mammalian target of rapamycin complex 2 (mTORC2) or deoxyribonucleic acid protein kinase (DNA-PK) for full activation [151,152]. The partial and full activation of Akt leads to its ability to modulate the functions of many substrates responsible for the acquisition of stem-like characteristics. The activation of Akt can be reversed by protein phosphatase 2 (PP2A, dephosphorylation of T308), PH domain and leucine rich repeat protein phosphatase 1/2 (PHLPP1/2, dephosphorylation of S473), and phosphatase and tensin homolog (PTEN, conversion of PIP_3_ to PIP_2_). Interestingly, although primarily localized to the cytoplasm and membrane-bound, recent work has shown that PTEN behaves as a scaffold protein in the nucleus to control genomic stability, splicing, apoptosis, and cell cycle progression [153,154,155], suggesting a non-enzymatic role of PTEN in regulating cancer stemness. However, this section will primarily focus on miRNAs and PTEN in the context of the PI3K/Akt signaling pathway.

Direct targeting of PI3K can have a major downstream impact on the acquisition of CSC characteristics. In support of this, targeting of PI3K, regulatory subunit 1 (PIK3R1) by miR-21 has been shown to reduce clonogenicity [85]. Furthermore, it has been shown that Akt is a direct target of several miRNAs. Akt exists in three different, closely related isoforms (Akt1, 2, and 3) that each have distinct biological functions. In breast cancer, miRNAs can target all three isoforms to regulate the acquisition of CSC characteristics. Direct targeting of Akt1 by miR-409-3p [131], Akt2 by miR-124 [102], and Akt3 by both miR-29c [89] and miR-93 [94] all result in a decrease in the ability of breast cancer cells to migrate and proliferate in soft agar. Lastly, miR-99 was found to be significantly downregulated in breast CSCs isolated by side population, and the re-expression of miR-99a significantly reduced the ability of MCF-7 and MDA-MB-231 cells to form spheroids [95]. It was later found that miR-99a was able to inhibit these CSC characteristics by directly targeting mTOR.

MiRNAs have also been shown to regulate the most prominent negative regulator of the PI3K pathway, PTEN. Bahena-Ocampo et al. found that miR-10b was significantly upregulated in two different CSC-enriched populations (CD44^+^ and EpCAM^+^) [77]. Stable expression of miR-10b increased the number of CD44^+^ and ALDH^+^ cells, as well as increased their ability to form spheroids, colonies in soft agar assays, and Akt signaling. Mechanistically, it was found that miR-10b was able to promote the acquisition of CSC phenotypes by downregulating PTEN expression [77]. Two separate studies also demonstrated that miR-20b promotes colony formation [80] and resistance to irradiation [81] through reduction of PTEN. In addition to regulating clonogenicity and radiation resistance, several papers have demonstrated that miRNAs promote drug resistance through targeting of PTEN. Wang and colleagues found that MCF-7 cells that are resistant to doxorubicin display increased levels of miR-21 [84]. The re-expression of miR-21 in MCF-7 cells resulted in the desensitization of these cells to doxorubicin, suggesting that miR-21 is critical for doxorubicin sensitivity. Further mechanistic studies revealed that miR-21 promotes chemoresistance by decreasing PTEN expression, and stable expression of PTEN alone is able to rescue this phenotype [84]. Furthermore, Ye et al. found that miR-221 promotes resistance to trastuzumab, a HER2-targeted breast cancer therapy, in SK-BR-3 cells, and the stable expression of PTEN in these cells was able to restore this therapy resistance [127].

### 4.5. NF-κB and TGF-β Signaling Pathways

NF-κB (nuclear factor kappa-light-chain-enhancer of activated B cells) refers to a family of transcription factors that homo- or heterodimerize to control transcription and modulate various cellular processes, such as survival and proliferation. This family consists of five transcription factors (RelA, RelB, c-Rel, NFκB (p50/p105), and NFκB2 (p52/p100)) [156]. Canonically, NF-κB transcription factors are maintained in inactive states by specific inhibitors (inhibitors of NFκB (IκB)) in the cytosol. However, once activated by ligands, IκB kinase (IKK) phosphorylates IκB, leading to the translocation of NF-κB proteins to the nucleus and activation of the transcription of target genes. Recent research has supported the function of miRNAs in processes that support breast cancer, such as proliferation, cell apoptosis, and inflammation, which are also regulated by NF-κB [157,158]. This has elucidated miRNAs that directly target genes central in NF-κB signaling cascades and contribute to the acquisition of stem-like phenotypes.

Wu et al. discovered that the transient transfection of miR-200b resulted in a functional decrease in the ability of SK-BR-3 and MCF-7 breast cancer cells to form colonies [119]. Furthermore, transient transfection with miR-200b was found to suppress IκBα phosphorylation, the expression of the p50 and p65 transcription factors, and NF-κB activity as defined by an electrophoretic mobility shift assay (EMSA). Using a bioinformatics approach, a putative miR-200b binding site in the 3′-UTR of IKKβ was identified. In a luciferase reporter assay, miR-200b was found to directly target IKKβ in breast cancer cells, and mutation of this binding site reduced this interaction. This demonstrates that miR-200b can directly target important players in the NF-κB pathway to reduce the functions that define CSCs. In support of this study, miR-708 has also been shown to target and reduce IKKβ expression [138]. Kumar and colleagues found that upon stimulation with glucocorticoids, miR-708 expression was significantly increased through activation by glucocorticoid receptor α. Transient transfection with a miR-708 mimic or with a glucocorticoid agonist not only resulted in decreased IKKβ expression, but it also reduced NF-κB activation and signaling in MCF-7 and MDA-MB-231 breast cancer cells [138]. Furthermore, miR-708 expression decreased the expression of typical CSC markers. Together, these data demonstrate that miRNAs can directly influence CSC characteristics through the NF-κB pathway.

The major nodes of NF-KB signaling can feed into and regulate TGF-β signaling [159]. While most work focuses on TGF-β activated SMAD signaling, Burk and colleagues found that members of the miR-200 family had significant effects directly on TGF-β [110]. They found that miR-141 and miR-200c strongly downregulated TGFβ2 and Zinc-finger E-box binding homeobox 1 (ZEB1), which is a well-known downstream transcription factor that promotes EMT. Interestingly, ZEB1 directly binds to and suppresses the expression of miR-141/-200c, suggesting that the loss of miR-141/-200c by ZEB1 expression results in a feed forward loop between TGFβ2 and ZEB1 that promotes tumor progression and recurrence.

### 4.6. BMI1 Signaling Pathway

B lymphoma Mo-MLV insertion region 1 homolog (BMI1) is a ring finger protein that is a major component of the Polycomb group complex of epigenetic regulators and is considered a proto-oncogene. As an epigenetic regulator, BMI1 predominantly resides in the nucleus where it can regulate multiple genes involved in stemness and self-renewal. In support of this, BMI1 expression has been shown to induce epithelial-to-mesenchymal transition (EMT, a normal developmental program), which is known to generate cells that gain many of the molecular and functional characteristics of CSCs in both cell-based assays and mouse models of cancer [23,160,161]. As EMT is well known to be regulated by miRNAs, such as the miR-200 family [57], it is not surprising that miRNAs directly target BMI1 to control the acquisition of CSC phenotypes.

Multiple studies have identified miR-200b/c as critical regulators of BMI1 signaling. For example, Polytarchou et al. isolated CSCs from transformed MCF10A cells, and, using a miRNA library, found that 16 miRNAs (including four members of the miR-200 family) significantly inhibited CSC growth and mammosphere formation [107]. The induction of miR-200c in a claudin-low mouse tumor model drove a reversal of EMT in vivo, promoted cell differentiation, and reduced the expression of BMI1 [123]. In support of EMT and BMI1 being critical for the acquisition of CSC phenotypes, miR-200c expression reduced the CD24^+^/CD29^+^ breast CSC population, and a limiting dilution assay further demonstrated that miR-200c reduced breast CSCs. In another study, miRNA profiling between CD24^−/low^/CD44^+^/lin^−^ CSC and CD24^+^/CD44^−^/lin^−^ populations revealed 37 differentially expressed miRNAs [122]. Of these differentially expressed miRNAs, the miR-200 family was found to be consistently downregulated in these and other tested human breast CSCs, as well as in mouse mammary CSCs. Among the miR-200 family members, only miR-200c was shown to directly target BMI1. Functionally, miR-200c expression suppressed colony formation, and re-expression of BMI1 rescued the phenotypes of miR-200c on colony formation [122].

In addition to miR-200c, miR-128 has also been shown to be important in BMI1-mediated acquisition of breast CSC phenotypes. Qian et al. found that miR-128 was the most frequently lost miRNA in breast cancer patients relative to adjacent normal tissue [105]. An enforced expression of miR-128 in MDA-MB-231 breast cancer cells reduced, while knockdown of miR-128 in MCF-10A enhanced the ability of these cells to form colonies in soft agar and matrigel, as well as sphere formation and the ability to form tumors in a limiting dilution assay. Additionally, the knockdown of miR-128 in MCF10A allowed these typically non-tumorigenic cells to form tumors, suggesting an increase in CSC characteristics. In support of this, this group also found that miR-128 stable expression reduced, while knockdown of miR-128 increased the percentage ALDH^+^ and CD24^−/low^/CD44^+^ cells. It was found that miR-128 enforced these phenotypes in part by the direct targeting of BMI1. This interaction was also confirmed in CSCs isolated from primary breast cancers [106], suggesting that this interaction is critical for CSC physiology.

## 5. Use of miRNAs as Biomarkers for Breast Cancer

MiRNAs have been identified and isolated from biological fluid, which are highly stable and are more resistant to environmental degradation. Furthermore, the correlation between miRNAs in the fluid and miRNA expression in tissue of origin [162,163], and that miRNAs can efficiently stratify cancer from normal tissue [164,165], suggests that miRNAs can be used as biomarkers for breast cancer diagnosis and prognosis. Typically, miRNAs are made within the cell, packaged into and then released into the biological fluid via extracellular vesicles [166,167], where they can directly regulate cancer progression in other cells. However, it has been recently suggested that miRNA biosynthesis can occur in extracellular vesicles in breast cancer [168], providing a new level of complexity. In either case, treatment with compounds that stop this process can prevent breast cancer progression by reducing miRNA-derived extracellular vesicles [169,170]. CSCs also secrete miRNAs in extracellular vesicles [171], suggesting that the successful treatment of breast cancer may lie in the ability to stop the secretion of extracellular vesicles containing miRNAs.

The use of miRNAs in extracellular vesicles as biomarkers has advantages for breast cancer. These include (1) it is a non-invasive technique to collect them, (2) the expression of some miRNAs can be identified in the blood before in the tissue, and (3) easy detection for monitoring post-therapy [172]. In support of this, miRNAs such as miR-1246 and miR-21 combined [173] and miR-373 [174] are just a few extracellular vesicle-derived miRNAs that have been shown to correlate well with breast cancer patient prognosis. However, more work is needed to develop more robust methods to identify and isolate extracellular vesicle miRNAs in biological fluids to improve patient outcome.

Additionally, freely circulating miRNAs have been isolated from blood and can be used as biomarkers for breast cancer. For example, circulating miRNAs, including miR-409-3p and miR-495 discussed above that regulate CSCs, were found to be significantly upregulated in the plasma of breast cancer patients relative to healthy control individuals [163,175], suggesting that these miRNAs can be used to identify breast cancer. Furthermore, Madhavan and colleagues used circulating miRNAs as a predictor for the circulating tumor cell (CTC, cancer cells found in the blood of patients with solid tumors, which display CSC properties and function as the seed for metastasis) status of patients with metastatic breast cancer [176]. This group found that eight miRNAs (miR-141, -200a/b/c, -210, -375, -203, and -801) were able to stratify CTC-positive from -negative patients, and six of eight were able to predict progression-free survival while all eight were found to be markers of overall survival. Lastly, CSC-regulating miRNAs miR-21 and miR-125b were found to be upregulated in the serum of breast cancer patients, and they are strongly, positively associated with the chemotherapeutic response of patients and distant metastasis-free survival [177]. These are a few examples of how miRNAs that generate or maintain CSCs can be used as a prognostic biomarker for breast cancer.

## 6. Targeting miRNAs to Develop New Breast CSC Therapies

Due to CSCs acquisition of chemo- and radioresistance, it is thought that targeting these CSCs offers the ability to overcome cancer progression and recurrence, ultimately leading to a better patient prognosis. In addition to the “age-old” strategies, or targeting the CSC-specific signaling pathways, monoclonal antibodies that target specific CSC surface markers have become an emerging technology for breast cancer therapy (reviewed elsewhere [73,178]). While the use of monoclonal antibodies has had great success in blood cancers [179,180], the use of this type of treatment has had limited success in solid tumors such as breast cancer. Major challenges exist in order for this to be a viable treatment for breast cancer including; (1) CSCs are typically present at a very low percentage of the tumor, (2) CSC markers overlap with other CSCs and even normal SCs, and (3) the absence of some markers are used to classify certain CSCs.

Instead, the use of miRNAs as a treatment option is another promising avenue, which can be used by itself or in combination with other treatments for CSCs. Increasing evidence indicates that the delivery of tumor suppressor miRNAs or oncogenic miRNA inhibitors has important therapeutic effects in pre-clinical models of breast cancer [181,182,183,184]. However, the main challenges in utilizing miRNAs in the clinic is the efficient delivery of these therapies. Therefore, much attention has been put on the development of innovative and specific delivery systems containing miRNAs. Non-viral-based delivery systems are at the leading front of miRNA therapeutics. However, due to a relatively lower efficiency than viral-based delivery systems, recent work has tried to increase efficacy by modifying either the particle size or the composition of the particle surface. For example, work from our group has shown that modification of the surface of a non-viral nanoparticle system enhanced the delivery of compounds that can be used to target properties of breast CSCs [185,186,187]. This same type of delivery system can be used to deliver miRNAs to elicit similar inhibitory effects on breast CSCs.

## 7. Summary and Perspectives

Although the existence of CSCs is able to explain many observations of breast cancer initiation and progression, there is still considerable research that needs to be done. Even though CSCs are observed in the clinic, the complete clinical relevance of CSCs is yet to be fully elucidated, especially in the case of breast cancer. However, therapies specifically targeting CSCs over SCs and normal differentiated cells is the key to the successful eradication of breast cancer. While we have a basic understanding of signaling pathways that underlie breast CSC physiology, a more refined understanding is needed. This will lead to better treatment strategies, such as dual antibody or drug–antibody treatments, and it affords patients a better prognosis. Furthermore, the fact that so many markers are used to identify breast CSCs adds even more complexity to an already complicated topic. The identification of reliable CSC markers, determining whether CSCs transition between the expression of these markers, and elucidating the effects on signaling pathways not only will better our understanding of CSCs but also of how miRNAs factor into these pathways.

**Figure 1:**
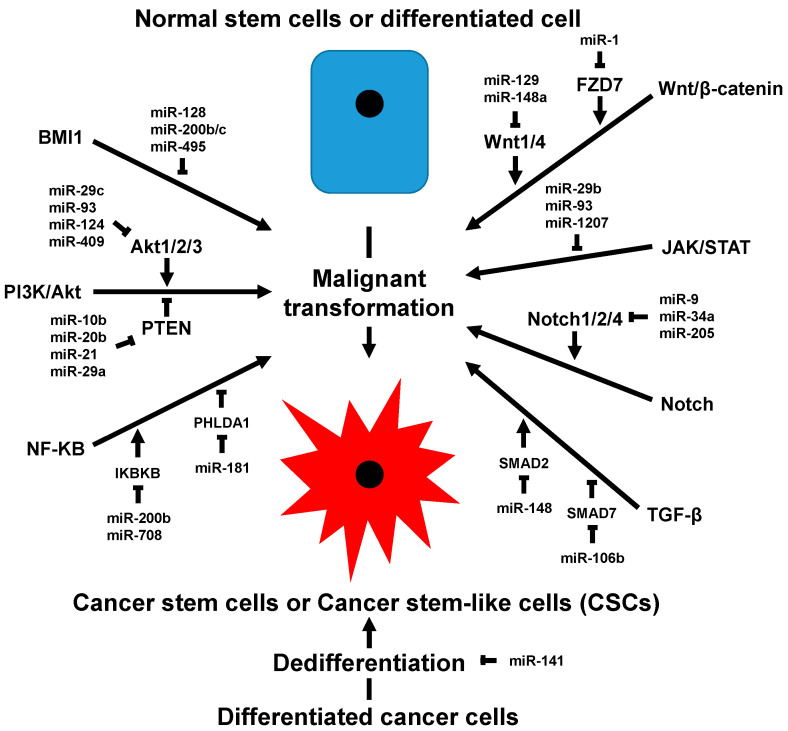
Summary of microRNA involvement in the generation and maintenance of breast cancer stem-like cells (CSCs). Generally, there are seven different pathways that have been shown to regulate breast CSC physiology. Activation of these pathways drives malignant transformation and helps to generate and/or maintain breast CSCs. Some representative microRNAs, targeting important players in each of these pathways, are shown. Although some studies have shown conflicting data, the literature generally agrees that microRNAs inhibit CSC formation and homeostasis.

In general, the data generally support the conclusion that the expression of miRNAs reduces the acquisition of CSC characteristics and reduces markers of breast CSCs, although the direct targeting of tumor suppressors, such as PTEN, demonstrate that miRNAs can promote CSCs (Figure 1). Future work will need to better identify direct targets of miRNAs and elucidate the mechanisms of how these miRNAs affect breast CSCs. In addition to determining miRNA targets and effects on signaling pathways, we also need to determine how to effectively deliver miRNAs to their target site. By overcoming this obstacle, we will be able to elucidate the role of miRNAs in mouse models of breast cancer, which will further their role as a promising therapeutic option. Although the clinical utility of ncRNAs in the clinic is limited, recent United States Federal Drug Administration (FDA) approval of patirisan [188] and PCA3 [189] ncRNAs show the potential of miRNA clinical applications in the future. Overall, the discovery of the central role of miRNAs within breast CSC signaling pathways not only provides new challenges but also offers opportunities for the development of novel therapeutic strategies to control breast CSCs.

## Figures and Tables

**Table 1:** Commonly used markers to identify breast CSCs in mouse and human cells.

Breast CSC Marker	Gene Name	Notes on Marker	Reference
ABCG2^+^	ATP-binding cassette superfamily G member 2	Membrane-associated protein; ATP-binding cassette (ABC) transporter; Xenobiotic transporter	[26]
ALDH1^br^	Aldehyde dehydrogensase 1	Cytosolic isozyme involved in alcohol metabolism; Involved in retinol metabolism	[18]
CD133^+^	Prominin 1	Pentaspan transmembrane glycoprotein; Often expressed in adult stem cells; Helps maintain stem cell properties	[27]
CD24^−/low^/CD44^+^	CD24: CD24 molecule	CD24: Sialoglycoprotein; Anchored to the cell surface via a glycosyl phosphatidylinositol (GPI) link; Modulates B-cell activation	[19]
	CD44: CD44 molecule	CD44: Cell-surface glycoprotein involved in cell–cell interactions; Receptor for hyaluronic acid but can also interact with osteopontin, collagen, and matrix metalloproteinases (MMPs)	
CD24^−/low^/CD44^+^/ANTXR1^+^	ANTXR1: Anthrax toxin receptor 1	ANTXR1: Type 1 transmembrane protein; Important for cell attachment and migration; Tumor-specific endothelial marker	[28]
CD24^−/low^/CD44^+^/SSEA-3^+^	SSEA-3: Stage-specific embryonic antigen 3	SSEA-3: Glycosphingolipid; Marker of mesenchymal stem cells	[29]
CD24^−/low^/CD49f^+^/CD44^+^/EpCAM^+^	CD49f: Integrin subunit alpha 6 (ITGA6)	CD49f: Member of the integrin alpha chain family of proteins; Associates with B1 or B4 subunits to interact with the extracellular matrix (ECM)	[30]
	EpCAM: Epithelial cell adhesion molecule	EpCAM: Carcinoma-associated antigen; Type 1 transmembrane protein; Mostly prominently expressed on epithelial cells	
CD24^+^/CD29^+^/CD49f^+^	CD29: Integrin beta 1 (ITGB1)	CD29: Member of the integrin beta chain family of proteins; Involved in cell adhesion and recognition in embryogenesis, homeostasis, tissue repair, and immune response	[31]
CD24^+^/Thy1^+^	Thy1: Thy-1 cell surface antigen	Thy1: Cell surface glycoprotein; Member of the immunoglobulin superfamily of proteins; Involved in cell adhesion and cell communication	[32]
CD29^low^/CD24^+^/CD61^+^	CD61: Integrin beta 1 (ITGB3)	CD61: Member of the integrin beta chain family of proteins; Involved in cell adhesion and cell-surface mediated signaling	[33]
CD49f^+^/DLL1^hi^/DNER^hi^	DLL1: Delta-like canonical Notch ligand 1	DLL1: Transmembrane ligand protein of Notch1-3 receptors; Important in mediating cell fate decisions during hematopoiesis and cell–cell communication	[34]
	DNER: Delta/Notch-like EGF repeat containing	DNER: Transmembrane protein; Activator of Notch1 signaling	
CD70^+^	CD70 molecule	Transmembrane protein; Belongs to the tumor necrosis factor ligand family	[35]
ESA^hi^/PROCR^hi^/SSEA-3^+^	ESA: Epithelial-specific antigen (also known as EpCAM)	ESA: Intimately involved in cell–cell adhesion and signaling	[29]
	PROCR: Protein C receptor	PROCR: N-glycosylated type 1 membrane protein; Receptor for and activates activated protein C	
GD2^+^	Ganglioside GD2	Glycosphingolipid; Relatively tumor-specific expression	[36]
Lgr5^hi^	Leucine rich repeat containing G protein-coupled receptor 5	Receptor for R-spondins and involved in the Wnt signaling pathway	[37]
MUC1^+^	Mucin 1, cell surface associated	Membrane-bound protein; O-glycosylated protein important for intracellular signaling; N-terminus functions in cell-adhesion; C-terminus functions in cell signaling	[38]
Nectin-4^+^	Nectin cell adhesion molecule 4	Involved in cell adhesion through trans-homophilic and heterophilic interactions; Single-pass type 1 membrane protein	[39]
PROCR^+^/ESA^+^			[40]
Sca1^+^	Stem cell antigen-1	Glycosyl phnosphatidylinositol-anchored cell surface protein; Regulates or coactivates cell signaling via receptor-ligand binding or other protein-protein interactions	[41]
Side population		Defined by the exclusion of Hoechst 33342 dye	[26,42]

**Table 2:** Experimental assays to measure phenotypes of breast cancer stem-like cells (CSCs).

Characteristic Increased in CSCs	Assay to Measure Phenotype	Reference
Clonogenicity	Soft agar colony formation	[44]
Sphere formation	Mammosphere formation assay	[45]
Tumor formation	Limiting dilution assay	[46]
Dye retention	Lipophilic dye retention (PKH26, PKH67, DiD, etc.)	[34,47]
Migration	Microfluidics-based migration device	[21]
Stemness markers	Fluorescence- or magnetic-activated cell sorting (FACS or MACS) with antibodies for stem markers identified in Table 1	[18,19]
Drug or radio-resistance	Drug curves, identification of the side population, radiation treatment/curves	[48,49]

**Table 3:** Targets of microRNAs (miRNAs) involved in breast cancer stemness.

microRNA	miRNA Acts as a Tumor Suppressor or Oncogenic miRNA	Target *	CSC-Associated Pathway Target is Involved in	Reference
miR-1	tumor suppressor	FZD7	Wnt/β-catenin	[74]
miR-7	tumor suppressor	SETDB1	JAK/STAT	[75]
miR-9	tumor suppressor	Notch1	Notch	[76]
miR-10b	oncogenic	PTEN	PI3K/Akt	[77]
miR-16	tumor suppressor	Wip1	Wnt/β-catenin	[78]
miR-18	oncogenic	SMAD7	TGF-β	[79]
miR-20b	oncogenic	PTEN	PI3K/Akt	[80,81]
miR-21	oncogenic	PTEN	PI3K/Akt	[82,83,84]
miR-21	oncogenic	PIK3R1	PI3K/Akt	[85]
miR-29a	oncogenic	PTEN	PI3K/Akt	[86]
miR-29b	tumor suppressor	TGFB2 and TGFB3	TGF-β	[87]
miR-29b	tumor suppressor	STAT3	JAK/STAT	[88]
miR-29c	tumor suppressor	Akt3	PI3K/Akt	[89]
miR-34a	not determined	WNT1	Wnt/β-catenin	[90]
miR-34a	tumor suppressor	Notch1	Notch	[91]
miR-34b	tumor suppressor	JAG1	Notch	[92]
miR-34c	tumor suppressor	Notch4	Notch	[93]
miR-93	tumor suppressor	Akt3	PI3K/Akt	[94]
miR-93	tumor suppressor	STAT3	JAK/STAT	[94]
miR-99a	tumor suppressor	mTOR	PI3K/Akt	[95]
miR-100	tumor suppressor	Plk1	Wnt/β-catenin	[96]
miR-100	tumor suppressor	mTOR	PI3K/Akt	[97]
miR-106a/b	tumor suppressor	STAT3	JAK/STAT	[98]
miR-106b-25	oncogenic	NEDD4L	Notch	[99]
miR-106b-25	oncogenic	SMAD7	TGF-β	[100]
miR-1207	oncogenic	STAT6	JAK/STAT	[101]
miR-124	tumor suppressor	Akt2	PI3K/Akt	[102]
miR-125b	tumor suppressor	CK2-a	Wnt/β-catenin	[103]
miR-126	tumor suppressor	PIK3R2	PI3K/Akt	[104]
miR-128	tumor suppressor	BMI1	BMI1	[105,106,107]
miR-129-5p	tumor suppressor	WNT4	Wnt/β-catenin	[30]
miR-130b-3p	tumor suppressor	DLL1	Notch	[108]
miR-139-5p	tumor suppressor	Notch1	Notch	[109]
miR-141	tumor suppressor	TGFB2	TGF-β	[110]
miR-141	tumor suppressor	STAT5A	JAK/STAT	[111]
miR-146a	oncogenic	NUMB	Notch	[112]
miR-146a/b	tumor suppressor	IRAK1 and TRAF6	NF-κB	[113]
miR-148	tumor suppressor	SMAD2	TGF-β	[114]
miR-148a	tumor suppressor	WNT1	Wnt/β-catenin	[115]
miR-181	oncogenic	ATM	TGFB	[116]
miR-181	oncogenic	PHLDA1	NF-κB	[117]
miR-181c	oncogenic	PTEN	PI3K/Akt	[118]
miR-200b	tumor suppressor	IKBKB	NF-κB	[119]
miR-200b	tumor suppressor	BMI1	BMI1	[107]
miR-200c	tumor suppressor	HIPK1	Wnt/β-catenin	[120]
miR-200c	tumor suppressor	PDCD10	PI3K/Akt	[121]
miR-200c	tumor suppressor	BMI1	BMI1	[122,123]
miR-205	tumor suppressor	Notch2	Notch	[124]
miR-205	tumor suppressor	ITGA5	PI3K/Akt	[125]
miR-221	oncogenic	ATXN1	Notch	[126]
miR-221	oncogenic	PTEN	PI3K/Akt	[127]
miR-222	oncogenic	PTEN	PI3K/Akt	[86,128]
miR-223	tumor suppressor	ITGA3	PI3K/Akt	[129]
miR-223	tumor suppressor	STAT5A	JAK/STAT	[129]
miR-301a	oncogenic	PTEN	PI3K/Akt	[130]
miR-409-3p	tumor suppressor	Akt1	PI3K/Akt	[131]
miR-448	tumor suppressor	NFKB	NF-κB	[132]
miR-495	tumor suppressor	BMI1	BMI1	[133]
miR-519d	tumor suppressor	STAT3	JAK/STAT	[134]
miR-520c	tumor suppressor	STAT3	JAK/STAT	[135]
miR-520h	oncogenic	DAPK2	PI3K/Akt	[136]
miR-544	tumor suppressor	STAT3	JAK/STAT	[137]
miR-708	tumor suppressor	IKBKB	NF-κB	[138]
miR-892b	tumor suppressor	TRAF2, TAK1, and TAB3	NF-κB	[139]
miR-3646	oncogenic	GSK3B	Wnt/β-catenin	[140]

*: As miRNAs can regulate multiple targets, it should be noted that other targets could also be involved in the phenotypes observed. The targets mentioned in this table are only the direct targets found by the authors in the cited manuscript.

## Data Availability

Not applicable.
